# A fine granularity based user collaboration algorithm for location privacy protection

**DOI:** 10.1371/journal.pone.0220278

**Published:** 2019-07-25

**Authors:** Bin Wang, Lei Zhang, Guoyin Zhang

**Affiliations:** 1 College of Computer Science and Technology, Harbin Engineering University, Harbin, PR China; 2 College of Information Science and Electronic Technology, Jiamusi University, Jiamusi, PR China; Victoria University, AUSTRALIA

## Abstract

As the location trajectory contains more spatial-temporal information about the user, it will be even dangerous for jeopardizing the privacy of the user. In order to cope with the correlation, an algorithm that utilizes the query division had been proposed. In this algorithm, random blocks of query context was used, so as the adversary was obfuscated and difficult to correlate the real result. However, this algorithm fails to dispose the size of each query block, as once same size blocks were obtained by the adversary continuously, so the adversary can regard them as blocks from the same query context, and then obtains the query context to correlate the discrete locations. In view of above conditions, in this paper we propose a fine granularity block division algorithm based on the conception of granularity measurement as well as granularity layer division, so with the help of collaborative users the location privacy of the user will be protected. In this algorithm, the query context will be divided into fine granularity size of information blocks that difficult to be distinguished with others, and then these blocks will be exchanged with other collaborative users to eliminate the difference in block size. In addition, as each block is divided into fine granularity size, the adversary will be difficult to correlate the discrete locations into location trajectory, so the location privacy will be protected. At last, through security analysis and experimental verification, this granularity indistinguishable algorithm is analyzed and verified at both theoretical and practical levels, which further demonstrate the superiority of the proposed algorithm compared with other similar algorithms.

## Introduction

At present, as the gradual improvement and development of technology on location based service (LBS), a vast of applications of this type of services are used in nearly all aspects of people's daily life and provide convenience for users. However, as this type of services usually utilize the real location of the user to provide precise feedback information such as navigation or restaurant searching, during the procedure of service providing the location privacy may be revealed and even result in some personal hazard such as tracking or robbing [1–3]. For example, once a user wants to drive to the nearest restaurant, she had to submit the query with the real location to obtain the feedback information to find the accurate road, during the service, her real location will be leaked to the server or other adversary and cause the location privacy leakage. Thus, the fear of location privacy leakage becomes an important problem that restricts the popularization of LBS and jeopardizes the development of relevant technologies.

In order to cope with the problem of location privacy leakage, a vast of location privacy protection algorithms were proposed, and these algorithms can be roughly divided into two main categories based on whether utilizes the central server (CS) [4–6]. The first type can be described as the centralized model, in this model a central server is used to provide location data obfuscation or concealment [7–10]. In this category, the location data is first sent to the CS and mixed with other locations or dummy locations then sent to the LBS server. As the real location is generalized into at least *k* similar locations, the adversary will be difficult to identify the real location and the location privacy will be protected. Algorithms of this category include the scheme of dummy location [7], the scheme of historical trajectory generalization [8], the scheme of similar trajectory generalization [9] as well as the scheme of attributes generalization [10]. However, as all the information was concentrated in the CS, it will be prone to become the attack point of adversaries or the bottleneck of privacy protection service. So the researcher has to consider utilizing another category without CS to provide privacy protection service, and utilizes disperse collaborative users to achieve privacy protection.

The other type of protection algorithms can be described as the distributed model, and this model just utilizes the devices of collaborative users to dispose the location data and achieves location data obfuscation or concealment [11–19], others include the schemes of encryption[20–22]. Algorithms of this category include the scheme of information topological transitive collaborative users selecting [11], the scheme of utilizing the cache of collaborative users [12, 18], the scheme of trajectory collaboration [19] as well as the scheme of query block randomly exchanging [16, 17]. The superiority of this category did not only be reflected in dispersing the disposing cost to collaborative users, but also been reflected in achieving the location privacy, the query privacy as well as the personal privacy simultaneously. However, although the query block randomly exchanging is better to other schemes in resisting the correlation attack, it also existing the failure in that the block size can be used to correlate the real location. As during the procedure of query block exchanging, the size of each collaborative user will be different to each other, and the difference of block size can be used to identify the discrete locations and correlate them into location trajectory.

As the superiority of distributed model is obviously, in order to reserve the ability of providing location privacy, query privacy and personal privacy simultaneously, and cope with the attack of correlation of block size difference, in this paper a fine granularity block division algorithm based on the conception of granularity measurement [23] as well as granularity layer division [24] is proposed, and utilize this algorithm to make up the deficiency of similar algorithms.

The rest of this paper is organized as follows. Firstly, we introduced the system architecture and basic idea in Section 2. Section 3 presents the algorithm based on the fine granularity information. Then we show the results of experimental with cause analysis in Sections 4. Finally, we draw conclusions and future works in Section 5.

## Preliminaries

### System architecture

**Fig 1:**
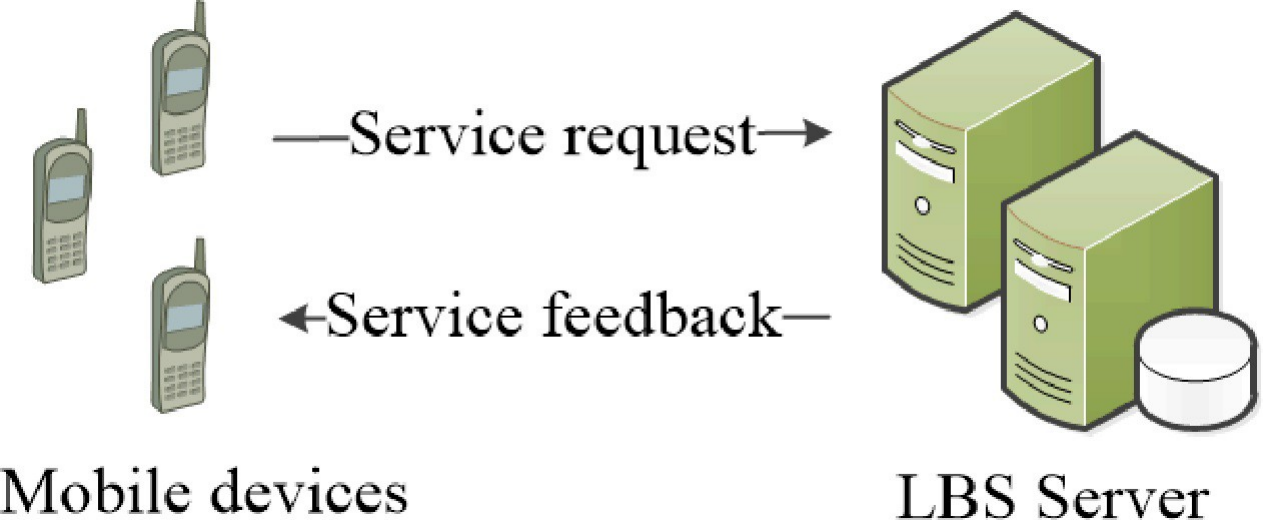
The system architecture of double layer.

In general, the system architecture of privacy protection algorithms can be divided into two main categories [1]. The first one is the centralized architecture model and the other one is the distributed architecture model. As the user collaboration algorithm was mainly designed to cope with the problem of attack point and service bottleneck, this type of algorithms usually employs the distributed architecture, and this architecture does not need the central server. For the architecture of distributed model, two entities are involved. The first one is the moving user, which refers to the user who equipped with the GPS device and can communicate with each other. The other one is the service provider such as LBS server, and this entity can provide location based service with the location that the user submitted. Therefore, the architecture of our proposed algorithm can be described as the double layer system architecture shown in Fig 1. In this Fig, two main entities are mobile users and LBS server. The mobile users mainly refer to the user who utilizes the LBS when moving in the road and at the same time the user also needs the service of privacy protection. The entity of LBS server can be seen as the LBS provider, this entity can provide LBS for the user who requests for the result of LBS, but on the other side this entity will be also curious about the privacy of the user, and may even reveal the privacy of the user due to the benefit of commerce or broken by the adversary. Thus, this entity is usually considered as the semi-trusted entity, due to the fact that it can provide LBS and may also jeopardize the personal privacy of the user.

### Privacy threat

As the LBS server usually considers as the semi-trusted entity and may reveal the privacy of the user due to others, the privacy threat was considered comes from this entity. Suppose that a query message of the user can be denoted as M={L(X,Y),Q}, where(*X*,*Y*) denotes the position coordinates or longitude and latitude, *Q* denotes the query context or interest point, so *M* can be seen as a piece of message constituted by the location information and query context. Once the query message was divided into several blocks of the same size, the information of each block can be denoted as $M'=\{m_{1},m_{2},\ldots ,m_{k}\}$, where *k* is the number of message blocks according to the anonymous value. Therefore, the size of each block can be denoted as Size(*m*_1_) = Size(*m*_2_) =,…,Size (= *m*_*k*_), if these blocks exchanged with other collaborative users and then sent to the LBS server, the gained message of the LBS server will be from different users and can be denoted as $M{'}_{i},0\leq i\leq k$. However, as the query context from different users will be various, the size of blocks will be different from each other and can be distinguished by the adversary, as the message of blocks satisfy $m_{1}^{i}\neq m_{1}^{j}$, which means the adversary can utilize the difference of block size to clustering them into different types, and then utilizes them as background knowledge to identify the real location and correlates the location trajectory.

Thus, based on above analyses, the adversary can utilize the clustering to identify the query context with the difference of block sizes. Suppose that the set of blocks about a query context can be denoted as $U=\{x_{1},x_{2},\ldots ,x_{n}\}$, then the result of at least *k* gathered types can be denoted as $C=\{\omega_{1},\omega_{2},\ldots ,\omega_{k}\},1\leq k\leq n$, if there exists $\overset{k}{\underset{i=1}{\cup }}\omega_{i}=U$ that satisfies $\omega_{i}\neq \phi ,i=1,2,\ldots ,k$ and $\omega_{i}\cap \omega_{j}=\phi ,i,j=1,2,\ldots ,k(i\neq j)$, the adversary can identify the specified blocks from the set of blocks, which means the adversary can utilize the clustering to identify the sent blocks with the difference of block size and then obtain the location privacy.

### Protection conception and basic idea

**Fig 2:**
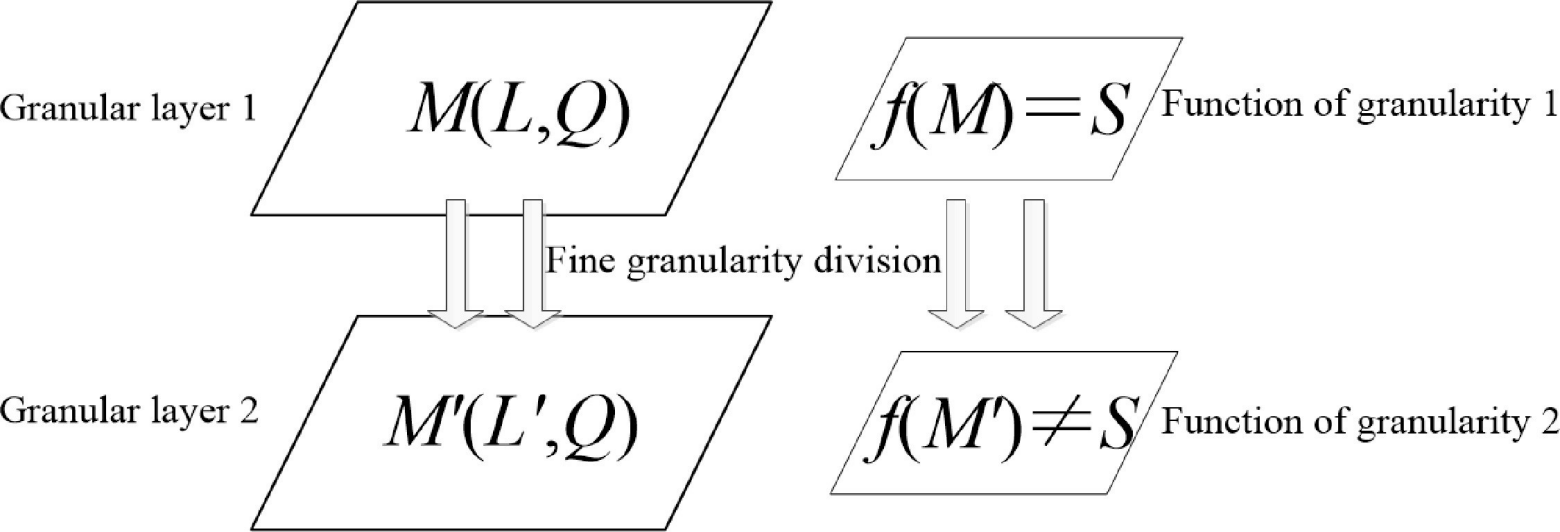
The fine granularity division of information with granular layer.

As the adversary can utilize the clustering to gather the difference of block size and identify the query context, the most effective method is to make the size equal to each and difficult to be distinguished, so the gathered set of each type satisfies $\omega_{i}\text{=}\omega_{j},i,j=1,2,\ldots ,k(i\neq j)$. In order to accomplish this purpose, the query context can be divided into fine granularity, so as the clustered set difficult to express the difference and difficult to be clustered into various types. Thus, based on above conception, we can utilize the criterion of different granular layers [24] in refining to establish the fine granularity query context blocks, and the procedure can be described in Fig 2.

In this Fig, M(L,Q) denotes the set of submitted query context blocks, function f(.) is the graining operation for each block in the set of submitted query context, *S* is the real information about the user. M'(L',Q) is the set of grained result for each block. Then with the help of fine granularity division, the query context blocks cannot be clustered and used to identify the real information with function f(.), so with the block size of clustered result, an adversary is difficult to identify the query context. Accordingly, the location privacy of the user will be protected.

## The protection algorithm based on the fine granularity information

As we had mentioned in section 2, the proposed protection algorithm based on the fine granularity information contains two main phases. The first one is the phase of query context fine granularity division, which used to divide the query context. The other phase is the information randomly exchanging, which used to exchange query information with other collaborative users and achieves the location and query anonymity. Then after above two phases, we provide a formal analysis for the security of proposed scheme.

### The procedure of fine granularity information division

In order to measure the granularity degree of divided query context blocks, this paper utilizes the metric used in literature [23]. In this paper we utilize GM(.) to denote the granularity degree of divided query context blocks, then the degree can be denoted as

$$GM(\pi )=\sum_{i=1}^{m}\frac{\left|X_{i}\right|}{\left|U\right|}\log|X_{i}|$$ (1)

Where $\pi =\{X_{1},X_{2},\ldots ,X_{m}\}$ denotes the division for the set of query contexts *U*, *X*_*i*_ is a subset of *U*. Once the block of a query context is divided into the fine granularity, which means each granule denotes the set of single point, in this condition GM(π)=0. Otherwise, if the size of granularity is the biggest granularity, the block will denote the whole query context and satisfies GM(π)=log|U|. Thus, in order to obfuscate the real block of the user, before exchanging the query block, each query context of collaborative users will be divided into fine granularity, so the size of block that submitted to the LBS server will be equal to each other and difficult to distinguish, and then the adversary will be difficult to identify the blocks of query context and more difficult to obtain the location privacy. The procedure of how to divide the size of query context into fine granularity can be described as algorithm 1.

Algorithm 1: The fine granularity division of query context

1) Input: The query information *M*
2) M'=M;
3) while (GM(M')≠0)
4) divides *M*';
5) end
6) Output: The divided query information *M*' in fine granularity

Once the query context is disposed by algorithm 1, the blocks for exchanging with collaborative user will be prepared, and then based on the block exchanging algorithm shown in literature [7], each user will have the fine granularity block that can be submitted to the LBS server, and with these blocks the adversary cannot identify the real query context easily. The procedure of disposing fine granularity block exchanging is described in algorithm 2.

Algorithm 2: The process of fine granularity information randomly exchanging

1) Input: M_a_; //The fine granularity information of other users
2) Input: n, u_num; //The number of users in current region
3) Initialization: Mexc = Null;
4) if(There is no granule in this granular layer)
5)   return;
6) else
7)   for(i = 1;i< = u_num+1; i++)
8)     n = M^i^; //The number of granules
9)     if(n>1)
10)       M_exc_ = M_exc_+M^i^_ran_; //Increase the random number
11)     else
12)       continue;
13)     end if
14)   end
15) end if
16) exchange M_exc_ with other collaborate user;
17) Output: M_exc_

After being disposed by algorithm 1 and algorithm 2, the blocks query context that sent to the LBS server will be dispersed to at least *k* locations and contains *k* different query types. In addition, the blocks in each set will be similar with each other in block size, and the adversary will be difficult to identify the query context with the difference of query block size, so the location privacy of the user will be protected. Based on above two algorithms, we can see that there are just one loop computing in each algorithm, so the time complexity can be summarized as O(n)+O(n)=O(n), which means the proposed protection algorithm can be finished no more than O(n).

### Security analysis

The security of our proposed algorithm depends on the uncertainty of correlating the block information with user's location and the uncertainty of the adversary successfully identify the real block with block size, so the security of our proposed algorithm can be inferred in the following two aspects. The first aspect is the uncertainty of correlating the block information with user's location. In our proposed algorithm, the message sent to the LBS server is divided and sent by at least *k* collaborative users in at least *k* different locations. In addition, the block set of query context will also contain at least *k* blocks of different contents. Therefore, the success probability of an adversary utilizes the block to correlate a real location will be equal to $1/k^{2}$. Once the anonymous value *k* is a value that large enough, it means the value of success probability of the adversary will be very small, and in this condition the location privacy of the user will be protected. For the aspect of uncertainty of the adversary successfully identifies the real block with block size. As all the collaborative users have divided their blocks of query information into fine granularity, the block will be similar with each other in block size and satisfy GM(M')=0. As a result, for at least *k* sets of clustering results that the adversary can obtain, the query context $M_{k}^{'}$ satisfies $C=\{\omega_{1}^{i},\omega_{2}^{i},\ldots ,\omega_{k}^{i}\},1\leq k\leq n,1\leq i\leq k$, where $\omega_{k}^{i}\neq \phi$ and $\omega_{i}\cap \omega_{j}\neq \phi ,i,j=1,2,\ldots ,k(i\neq j)$. It can be also explained as that if the adversary utilizes data cluster for the received blocks of query context, each set can be correlated with each other and all the clustered sets will be similar to each other. Thus, for the adversary, the probability of accurately identify the real user will be no more than 1/*k*, and the value will be even lower as the number of blocks also been considered and the value will be lower than $1/k^{2}$, so it will be difficult to correlate any set to any randomly chosen user and identify the real location of this user. Accordingly, the location privacy of the user will be protected.

## Experimental verification

In order to evaluate the performance of our proposed algorithm in the ability of privacy protection and the efficiency of algorithm execution, in this paper this algorithm is verified in simulation environment and compared with some similar algorithms. All the simulation experiments are performed in the laptop with Intel core i5, 4 GB memory and windows 7 operation system, and utilized Matlab 2017a to carry out the verification. The data set utilizes the real data collected from the traveling trajectory of Beijing taxi, and the algorithms used to compare contain the information topological transitive collaborative users selecting algorithm OQEU [11], the randomly exchanging information block algorithm RQBE [16], the result caching algorithm CAQBE [18] as well as the random walking algorithm RWBC [13]. The verification criterion reflects in the ability of privacy protection and the efficiency of algorithm execution. For the criterion of ability of privacy protection, the indistinguishable of block size with fine granularity and the uncertainty of an adversary identify a user is utilized. For the criterion of efficiency of algorithm execution, the running time and communication cost are utilized as metric to measure the performance. At last, the simulation results with corresponding analysis will further demonstrate the superiority of our proposed algorithm by the comparison with other similar algorithms.

**Fig 3:**
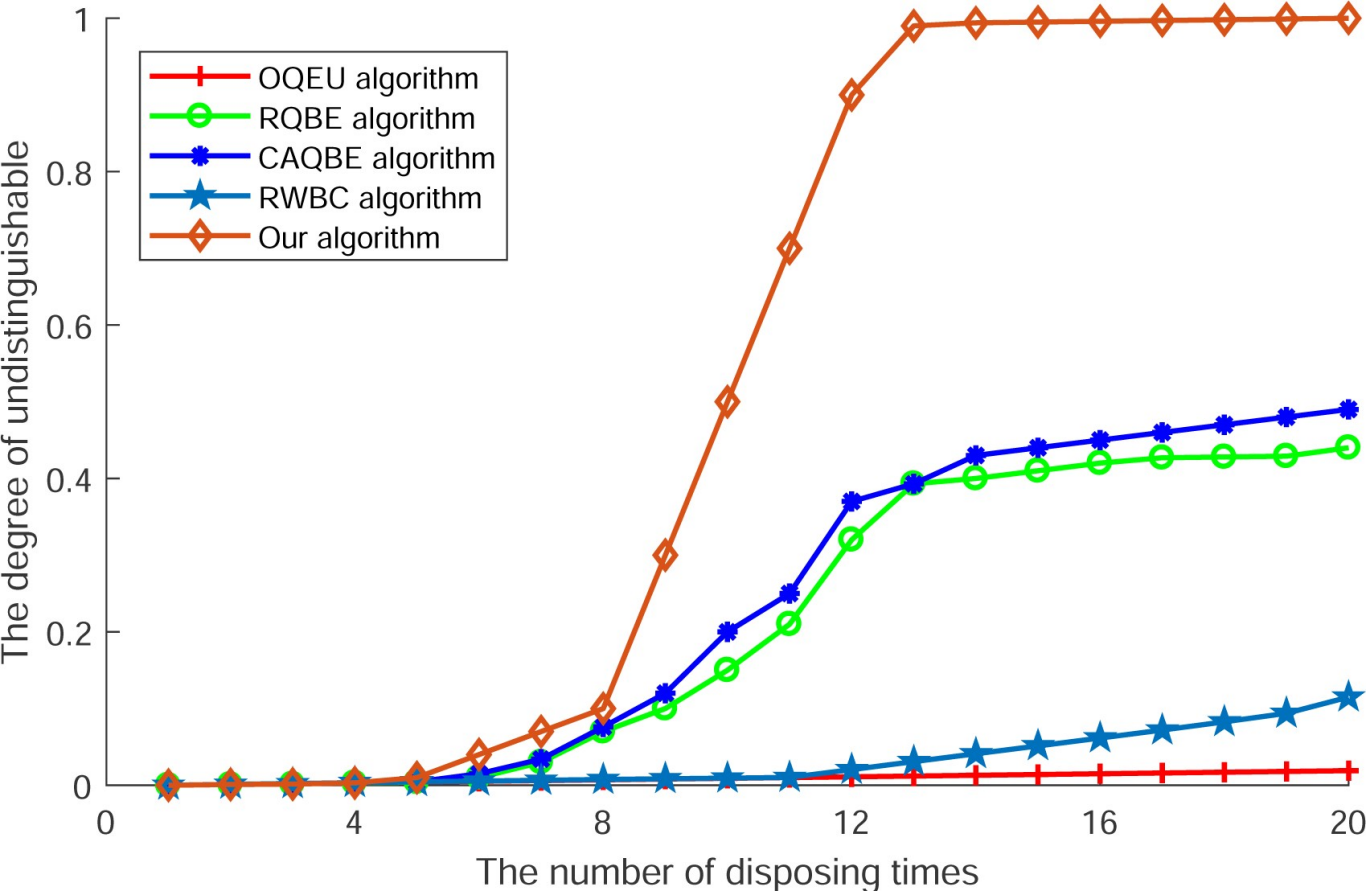
The degree of undistinguishable.

Fig 3 shows the degree of undistinguishable of block size with fine granularity. In this Fig, we can see that along with the increase of disposing times in devices of users, the degrees of all algorithms are ascending. However, among these algorithms, the degrees of OQEU and RWBC are lower than other algorithms, as these two algorithms mainly utilize the strategy of transmitting complete information in each collaborative user to choose the anonymous users, and the transmitted query context does not be divided into block or fine granularity, so the disposition degree for block size of undistinguishable is lower. For the other two algorithms RQBE and CAQBE, as these algorithms mainly utilize the exchange of a random number of blocks to search for collaborative users, the size of blocks of query context will be different to each other due to the variety of anonymous values. In addition, if the anonymous value is big enough, the query context will be divided into small size of blocks and if the other collaborative user also requires a high level of privacy protection and utilize a big anonymous value, the blocks of these users will be difficult to distinguish, so there will generate obvious transition of increasing in the degree of blocks undistinguishable. However, as difficult for all collaborative users choosing the biggest anonymous value, this degree of undistinguishable is limited and difficult to satisfy the most degree of undistinguishable. At last, as the propose algorithm is designed to cope with the difference of block size and in this algorithm the granularity measurement as well as granularity layer division are used to dispose the blocks of query context into fine granularity, so the set of submitted blocks are equal to each other and difficult to be distinguished. Accordingly, this algorithm can provide the highest degree of undistinguishable of block size with the increasing of disposing times and provide a better performance in undistinguishing.

**Fig 4:**
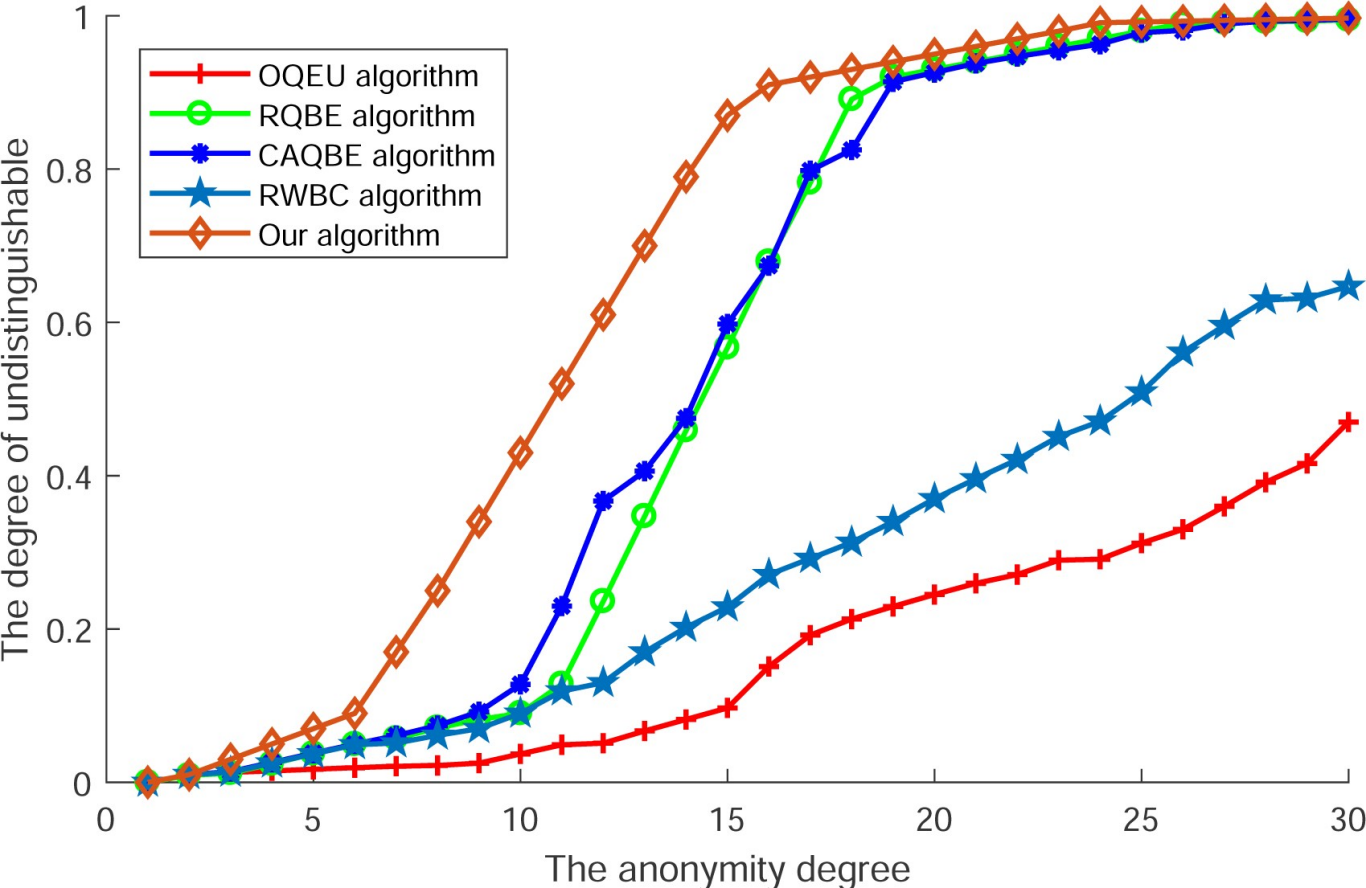
The degree of uncertainty of an adversary identify a user.

Fig 4 shows the degree of uncertainty of an adversary identifies a user. In this Fig, we can see that along with the increase of anonymity degree, degrees of uncertainty of all algorithms are ascending. However, among these algorithms, the degrees of OQEU and RWBC are lower than other algorithms and no matter the increasing of anonymity degree and their degrees of uncertainty are difficult to reach the highest value. The reason for this condition can be ascribed as that these algorithms do not dispose the message of query context into blocks but just transmit it to select collaborative users, so the degree of uncertainty is lower than other algorithms and the query context can be used to correlate the real location into location trajectory to infer the personal privacy. For algorithms of RQBE and CAQBE, as these algorithms utilize the randomly exchanging of query blocks, so when the degree of anonymity ascending to a certain value the degree of uncertainty will increase to the highest value and the adversary will be difficult to identify the real user. At last, as the algorithm proposed by this paper has divided the message of query context into fine granularity, which makes the blocks that exchanged with collaborative users are similar to each other in block size, so in this condition the correlation between location and block of query context can be extremely concealed and then at the condition of smaller degree, the degree of uncertainty will increase to the highest value.

**Fig 5:**
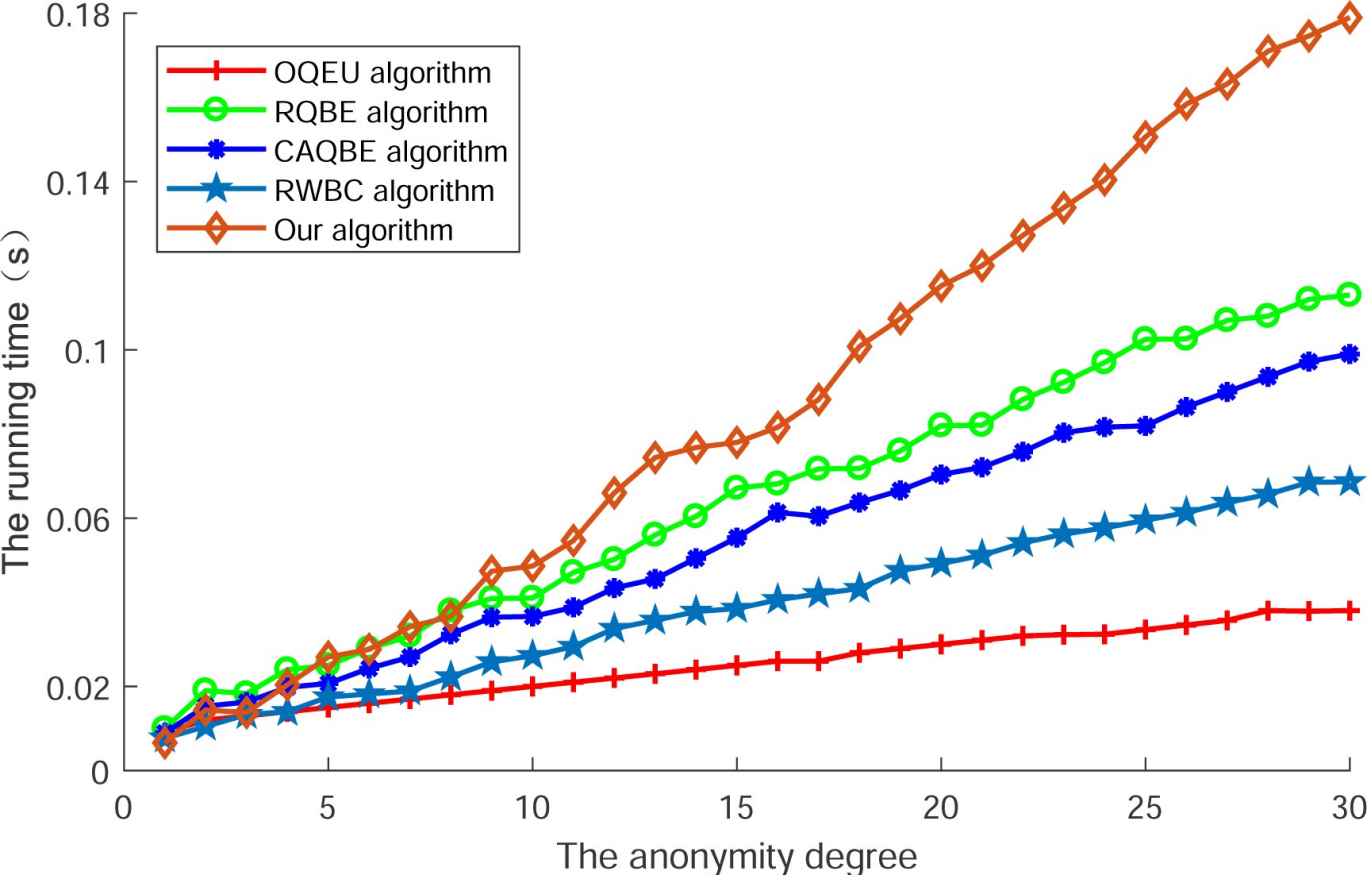
The running time of each algorithm.

Fig 5 shows the running time of our proposed algorithm compared with other algorithms. In this Fig, we can see that along with the increase of anonymity degree, running time of all algorithms is ascending. However, among these algorithms, the running time of OQEU and RWBC are lower than other algorithms, as the procedure of these algorithms just contain the disposing of information transformation and collaborative users selection, so the less process leads to the lower running time, but the lower level of privacy protection. For other algorithms such as RQBE and CAQBE, the running time is higher than above two algorithms, as the procedure of dividing information and exchanging with users to select the collaborative user had consume the running time. At last, the running time of our proposed algorithm is the highest, as this algorithm has to divide the message of query context into fine granularity that smaller than the randomly divided blocks, so it's running time is higher than all the compared algorithms, but the running time is under 0.18 seconds as the anonymity degree reaches 30, which is not higher than the waiting time the user difficult to bearing.

**Fig 6:**
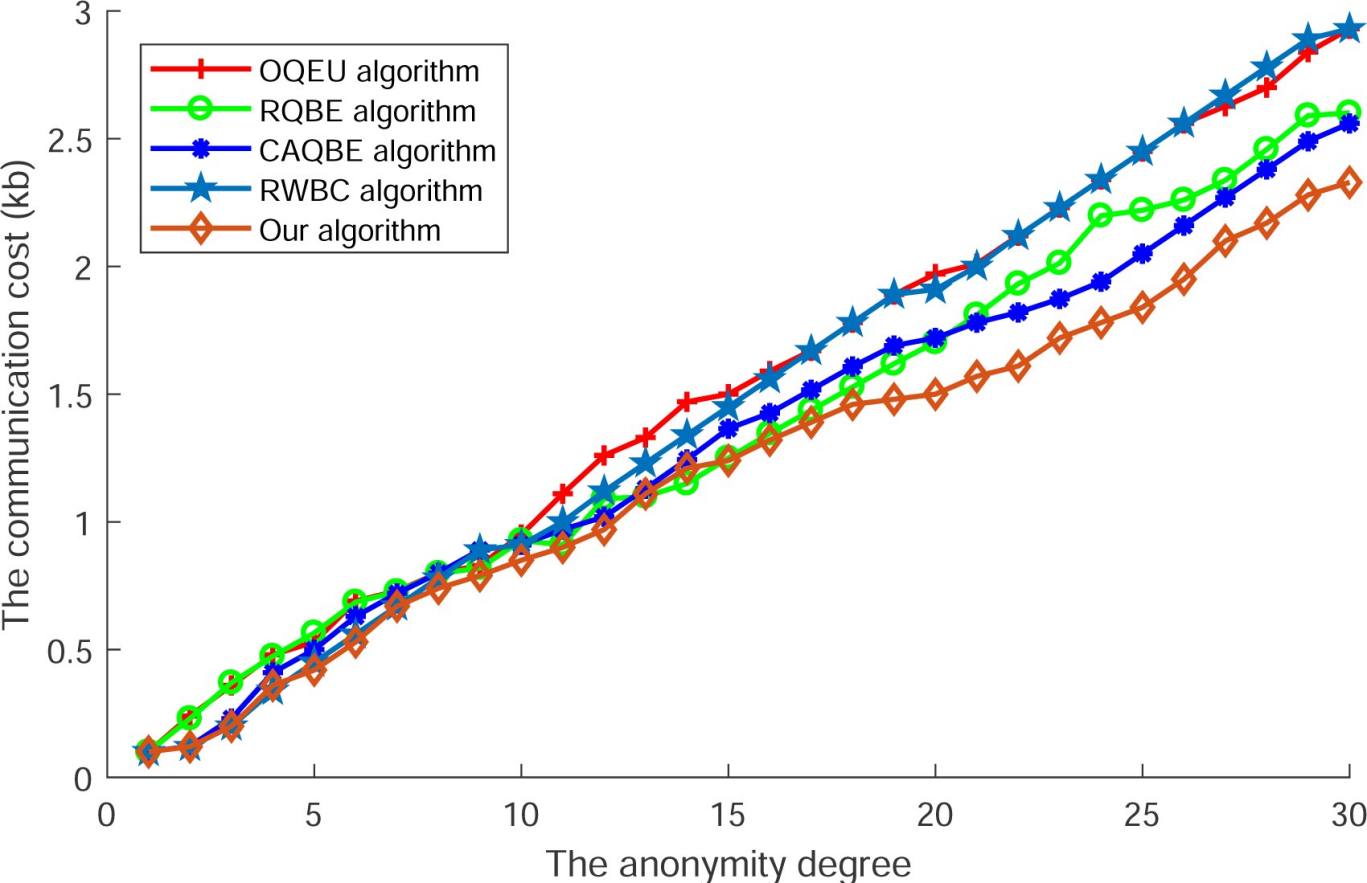
The communication cost of each algorithm.

Fig 6 shows the communication cost of all compared algorithms. In this Fig, we can see that along with the increase of anonymity degree, the communication cost of all algorithms is ascending. However, among these algorithms, the communication cost of OQEU and RWBC are higher than other algorithms, as these algorithms had to transmit the whole message to each other to select the collaborative users, so the user in the latter phase will send the whole set of nearly all messages of the user group and the communication cost between each user is higher. For other algorithms such as RQBE and CAQBE, the communication cost is lower, as these algorithms just exchange the query block to select collaborative users and the smaller size of block will reduce the communication cost used in blocks exchanging. At last, the communication cost of our proposed algorithm is the lowest, as the fine granularity of query context is smaller than the randomly divided block, so among the process of blocks exchanging, the cost of this algorithm is the smallest.

In conclusion, we can consider that the algorithm proposed in this paper is better than other compared algorithms in the performance of the ability of privacy protection and the efficiency of algorithm execution, so this algorithm has a better value in practical and promotion than other algorithms.

## Conclusion and future works

As the prevalence of location based service, location privacy had turned to be one of the most important issues and paid close attention. The algorithm of user collaboration is an efficient method used to cope with the service of bottleneck as well as the problem of point of attacks. However, algorithms of this type are all failure to refine the block size and the difference of block size can be used to identify the query context and then inferred to correlate discrete locations into location trajectory. In order to cope with this problem, in this paper we proposed a fine granularity block division algorithm based on the conception of granularity measurement as well as granularity layer division. Then with the divided fine granularity size of blocks, the adversary will be difficult to distinguish whom the block he grasped belongs to and difficult to identify the location, no matter utilizes the size clustering granularity or other correlation methods, so the location privacy of the user will be protected. At the same time, this algorithm also maintains the superiority of personal anonymity in the algorithm of exchanging random numbers of blocks, and all users in this algorithm can also enjoy the service of personal anonymity. However, as results shown in comparison with other collaborative user algorithms, this algorithm also restricted by the number of collaborative users and a lower number of users will lead to the failure of algorithm execution, so the future work will focus on how to descend the affection of collaborative users and how to ascend the ratio of algorithm success execution.

## Supporting information

S1 Table (XLS)
